# Circulating plasma levels of miR-106b-5p predicts maximal performance in female and male elite kayakers

**DOI:** 10.3389/fspor.2023.1040955

**Published:** 2023-02-14

**Authors:** Irene Torres-Aguilera, Paola Pinto-Hernandez, Eduardo Iglesias-Gutierrez, Nicolas Terrados, Manuel Fernandez-Sanjurjo

**Affiliations:** ^1^ Department of Functional Biology (Physiology), University of Oviedo, Oviedo, Spain; ^2^ Health Research Institute of the Principality of Asturias (ISPA), Oviedo, Spain; ^3^ Unidad Regional de Medicina Deportiva, Avilés, Spain

**Keywords:** female athlete, male athlete, circulating microRNAs, physical performance, exercise biomarkers, molecular response to exercise

## Abstract

**Introduction:**

Plasma miR-106b-5p levels have been described as an exercise performance predictor in male amateur runners, although no information is available about female athletes. The aim of this study was to analyze the predictive value on sports performance of plasma miR-106b-5p levels in elite female and male kayakers at the beginning and at the end of a training macrocycle, as well as the potential underlying molecular mechanisms using an *in silico* approach.

**Materials and Methods:**

Eight elite male (26.2 ± 3.6 years) and seven elite female (17.4 ± 0.5 years) kayakers from the Spanish national team. Two fasting blood samples were collected, starting point of the season (A) and maximum fitness level (B). Circulating plasma levels of miR-106b-5p were analyzed by RT-qPCR. Maximal 500 m performance was recorded at B.

**Results and Discussion:**

miR-106b-5p levels had no differences between A and B neither in women nor in men. In men but not in women, miR-106b-5p levels showed a negative significant correlation with performance in B which highlights its predictive value for performance. However, in women, progesterone emerged as a determinant and the ratio miR-106b-5p/progesterone showed a significant negative correlation with performance. *In silico* analysis reveals potential targets in a number of genes of relevant to exercise.

**Conclusions:**

miR-106b-5p emerges as a biomarker of athletic performance in men and in women, if the menstrual cycle is considered. This highlights the need to analyze molecular response to exercise in men and women separately, and considering the stage of the menstrual cycle in women as a relevant factor.

## 1. Introduction

Exercise determines a complex cross-talk between tissues, with profound effects on gene expression (1). Circulating microRNAs (c-miRNAs) are intercellular communicators with a posttranscriptional negative regulatory role that have emerged during the last decade as biomarkers and regulators of exercise response and adaption (2). Some authors have described a strong relationship between exercise performance and both the baseline levels of certain c-miRNAs or their changes in response to acute aerobic exercise (3, 4). However, there are no data of a specific microRNA analysis comparing both sexes at the same conditions. MiR-106b-5p was validated as a predictor of maximal aerobic speed in a laboratory conditions test (4). Moreover, miR-106b-5p levels were downregulated in response just after a marathon (5) and also downregulated, 24 h after a marathon in male amateur runners (6). However, no changes were observed during a whole season in the same amateur runners (5). These standardized laboratory tests and competition findings are useful to evaluate amateurs’ response but practical data is necessary to meet the needs of elite sportsmen and women (7).

The aim of this study was to analyze the response of miR-106b-5p in both sexes with training and its relationship with exercise performance in kayakers.

## 2. Methods

### 2.1. Ethics statement

Experimental procedures were approved by the Research Ethics Committee of the Principality of Asturias, Spain (reference: 124/17). All participants gave written informed consent.

### 2.2. Subjects

Eight elite male athletes (26.2 ± 3.6 years) and seven elite female athletes (17.4 ± 0.5 years) from the Spanish national kayaking team were recruited. This group included several Olympic and world medalists and was homogeneous, not only in terms of performance level (best time 500 m in seconds male: 101.24 ± 1.02; female: 121.80 ± 3.80), but also regarding accommodation and, since they trained and competed to be part of the K4 boat that will eventually participate in the Olympics. In the group of female athletes none of them used oral contraceptives.

### 2.3. Procedures

#### 2.3.1. Sampling protocol

In a season training design, we have defined two fit peaks. First one is the most unfitted one, we collected the first blood sample in the firsts two weeks of training (point A). The second sampling point was defined by the main goal of the season, one week previous this competition was the second sampling point (point B).

#### 2.3.2. Performance test

Physical performance was measured in a training-controlled situation by a maximum test in 500 m with every single athlete in their own kayak at B (Tests Times in seconds: males 102.03 ± 1.72 and females 125.15 ± 4.07). This distance was selected because is the main objective to all athletes, both men and women.

#### 2.3.3. Blood sampling

Subjects had a blood sample taken in fasting state. Blood samples (<10 ml) were collected in vacutainers [No Additive (Z), Becton Dickinson, United States], stored at room temperature for at least 15 min to allow clot formation, and immediately centrifuged at 1500 g for 15 min at 10 °C. Serum samples were then aliquoted and stored at −80 °C for later analysis.

#### 2.3.4. miRNA analysis

Total RNA was isolated from 200 μl of serum using the miRCURY RNA isolation kit (Qiagen). For ulterior normalization, synthetic cel-miR-39-3p was added. The LNA Spike-in kit with synthetic RNA spike-in templates (UniSp2, UniSp4, UniSp5) (Qiagen) was used to monitor RNA isolation efficiency. For miRNA quantification, cDNA was synthesized using the universal LNA RT kit (Qiagen). Additionally, UniSp6 (Qiagen), was added to check for RT efficiency. For qPCR, cDNA was diluted 80× and 4 µl used in 10 µl qPCR reactions with miRCURY LNA SYBR Green (Qiagen) on a 7900HT fast Real-Time PCR System (Applied Biosystems). To discard the presence of nucleases, inhibitors or hemolysis, the miRCURY miRNA Quality Control PCR Panel (Qiagen) was used before miRNA analysis. Hsa- miR-23a-3p (seq. AUCACAUUGCCAGGGAUUUCC), hsa-miR-451a (seq. AAACCGUUACCAUUACUGAGUU) and hsa-mir-106b-5p (seq. UAAAGUGCUGACAGUGCAGAU) were analyzed with LNA primers (Qiagen). SDS v2.3 software was used for both the determination of the quantification cycle (Cq) and for melting curve analysis. The dCq(miR-23a-3p – miR-451a) method was used to confirm that none of the samples were affected by hemolysis (all samples had dCq value below 6). miRNAs were considered to be expressed when Cq < 37 or were detected with at least 5 Cq below the negative control. Normalization to miR-451a, because his stable values, was performed, representing the data as −*Δ*ct (8). GenEx software (MultiD Analyses AB, Sweden) was used for data processing and miRNA relative expression analysis.

#### 2.3.5. Pathway analysis

For each miRNA, experimentally validated targets were retrieved from miRTarBase v7 database. Pathway annotations for each gene were retrieved from KEGG pathways using Diana mirpath v3(9, 10). Thus, we obtained gene sets and metabolic pathways linked to miRNA targeting genes. The results output a log odds ratio for each interrogated gene set, along with raw and false discovery adjusted *p*-values. The data represented was number of genes per pathway. For tissue expression analysis was used miTED (11) and data were represented on RPM.

We used target mining analysis by miRWalk, where it defined a whole complete list of gene targets. With this database, we used Gene Ontology of protein classes and biological processes on Pantherdb 17.0 tool. All data represented was by number of genes target.

#### 2.3.6. Statistical analyses

Normality of variables was tested using Shapiro–Wilk's test. Male and female groups had nonsignificant *p*-values, so normality could be assumed. Data are expressed as mean ± SD. Associations between variables were analyzed using Pearson's correlation analysis. A multiple paired samples *T*-test was performed to compare A vs. B samples. *p*-values < 0.05 were considered significant. A customized R (www.r-project.org) function was used for all processes.

## 3. Results

### 3.1. No significant change of miR-106b-5p caused by training

Sexual hormones were measured at both sampling points with no differences (Table 1).

**Table 1:** Levels of sexual hormones in athletes at both sampling points.

Sampling point	A		B	
	Men	Women	Men	Women
Total Testosterone (ng/ml)	5.9 ± 0.8	0.4 ± 0.1	6.3 ± 1.3	0.5 ± 0.2
FSH(U/L)	—	3.4 ± 2.9	—	4.6 ± 2.3
LH (UI/L)	—	8.0 ± 5.3	—	8.9 ± 5.0
Estradiol (pg/ml)	—	56.3 ± 50.8	—	70.2 ± 72.0
Progesterone (ng/ml)	—	2.5 ± 5.1	—	2.4 ± 3.5

FSH, follicular stimulating hormone; LH, lutropin; —, not measured.

**Figure 1:**
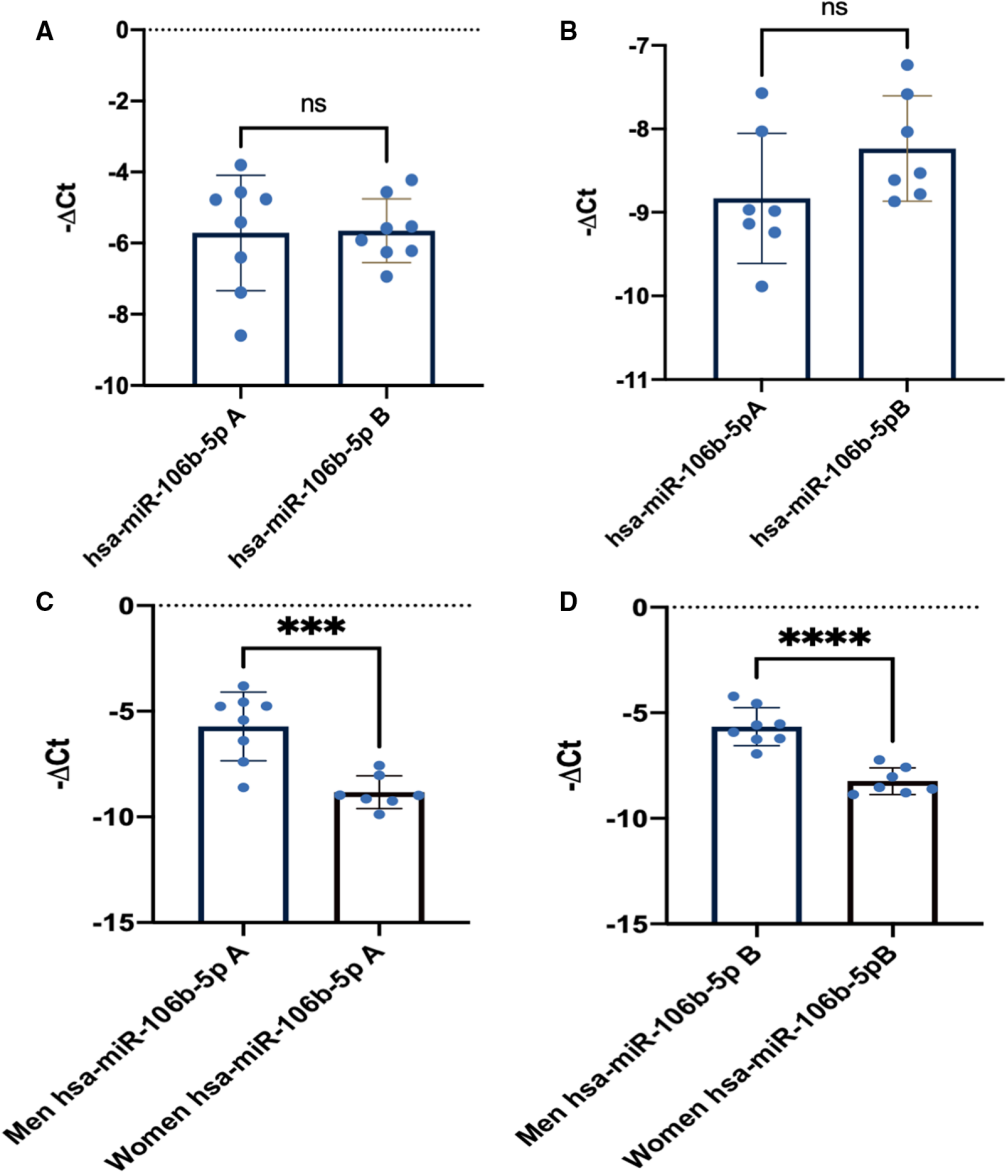
miR-106b-5p training response in men and women. (**A**) Mir-106b-5p men athletes’ response at point A and B. (**B**) MiR-106b-5p women athletes’ response at point A and B. (**C**) Men vs. women comparison at point A. (**D**) Men vs. women comparison at point B. Data are presented as means, single values and SD. Ns, nonsignificant changes A–B.

As it could be observed in Figures 1A,B, training did not change miR-106b-5p levels in elite athletes both in men and women. However, levels between sexes were different in both sampling points with a lower level in women than in men. Moreover, in Figures 1C,D, levels of circulating miR-106b-5p were significantly lower in women than in men in both sampling points.

### 3.2. A negative relationship between circulating miR-106b-5p levels and physical performance

**Figure 2:**
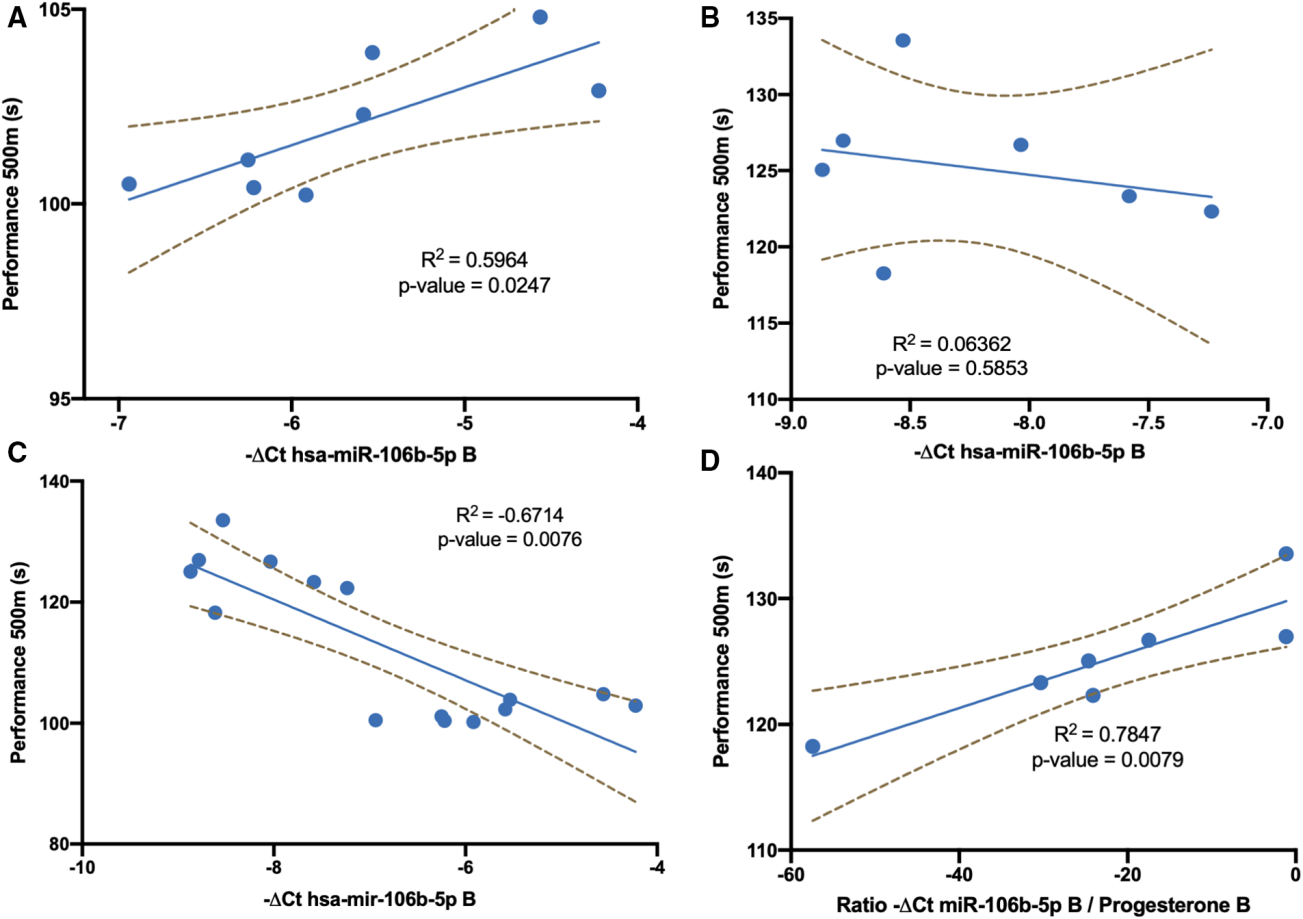
Linear regression analysis of exercise performance data. (**A**) Mir-106b-5p levels and exercise performance in men athletes at point B. (**B**) Mir-106b-5p levels and exercise performance in women athletes at point B. (**C**) Mir-106b-5p levels and exercise performance both in men and women athletes at point B. (**D**) Ratio of mir-106b-5p levels / progesterone and exercise performance in women athletes at point B.

We analyzed whether the miR-106b-5p response showed the same relationship with performance when considering men and women together or separately. A significant negative correlation was described between exercise performance and miR-106b-5p at B in male athletes (Figure 2A). No significant correlation was observed between mir-106b-5p levels and exercise performance in female athletes (Figure 2B). If we considered both sexes at the same time, the result was a positive significant correlation (Figure 2C). As could be observed in this figure, both groups must be considered separately. Furthermore, considering women and men together would fall into a methodological and statistical error by observing a positive correlation.

Since men and women behave differently, we analyzed the possible influence of hormonal changes during the menstrual cycle on this relationship. A tendency between progesterone levels and exercise performance seems to be established at point B (*p*-value 0.057, Pearson *r* 0.741). Moreover, if this hormone was taken into account to normalize miR-106b-5p levels a negative significant negative correlation was obtained (Figure 2D).

### 3.3. Target pathway analysis

**Figure 3:**
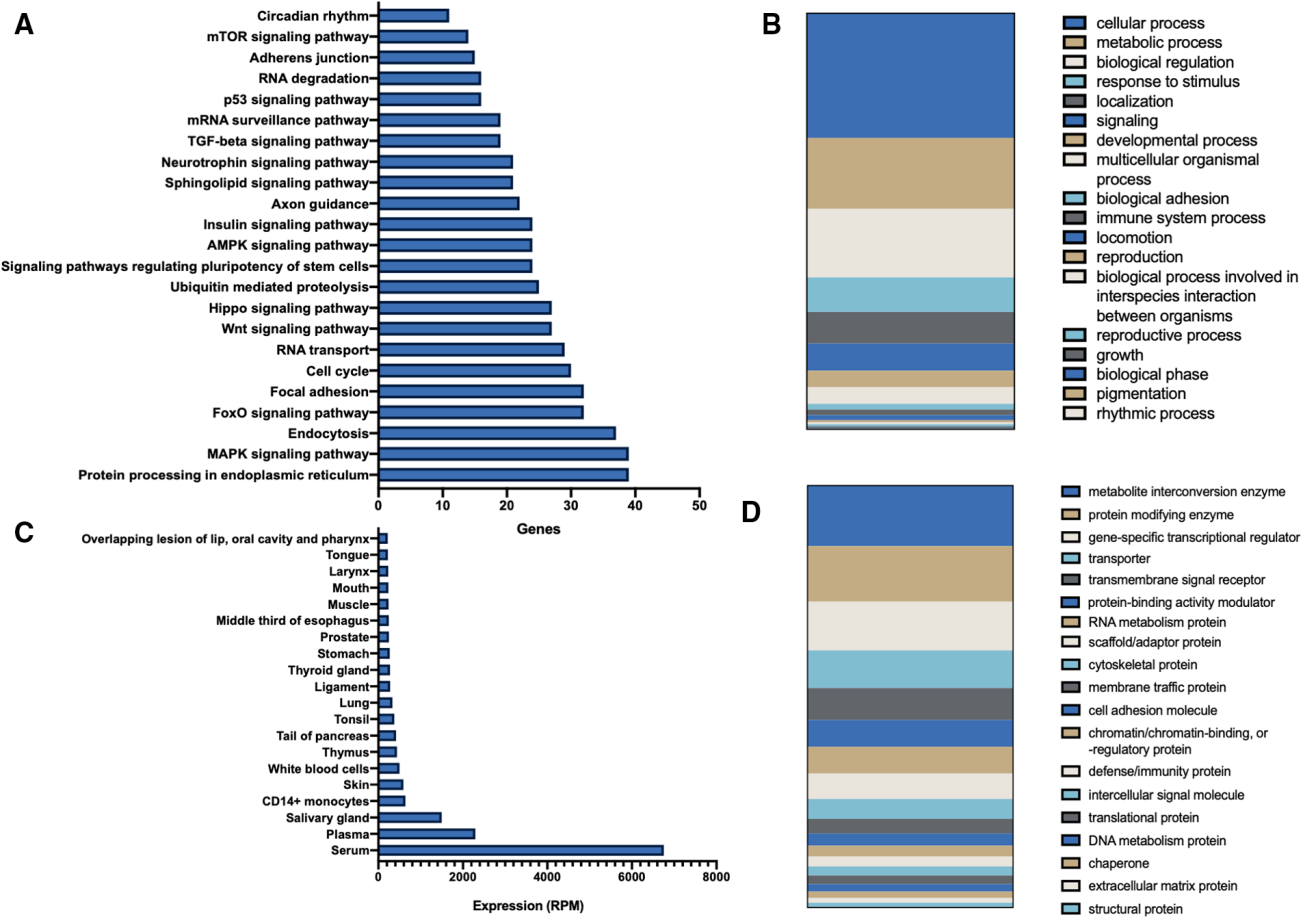
*In silico* analysis of mirR-106b-5p targets, functions and tissue expression. (**A**) KEGG analysis based on miRTarBase validated targets on miRpath v3. (**B**) miR-106b-5p miTED analysis for tissue levels. (**C**) Molecular process analysis of miR-106b-5p targets by mirWalk database and pantherdb tool. (**D**) Protein function analysis of miR-106b-5p targets by mirWalk database and pantherdb tool.

*In silico* analysis showed a strong relationship between mir-106b-5p and pathways related with exercise such as FoxO, Hippo, and AMPK signaling pathway (Figure 3A). Tissue-specific analysis showed that the main source of miR-106b-5p is found in blood and blood cells, but also in muscle and tendons (Figure 3B). With regard to biological processes, the main regulations are observed at the level of metabolic and cellular processes and biological regulation (Figure 3C). In the analysis of proteins, the main control over metabolite interconversion enzymes, which are decisive in the energy metabolism of exercise, must be emphasized (Figure 3D). Hydrolases were one of the main targets of the group metabolite interconversion, all of them related with catabolic processes and involved in exercise metabolism.

## 4. Discussion

Our results showed a specific response of c-miR-106-5p between sexes. In the case of male athletes, a clear negative relationship was established between c-miR-106-5p expression and exercise performance. The joint analysis of women and men may mask a different or asymmetrical behavior in each sex, which would affect both the interpretation of the results and their practical application. As our results show, we observed a significant difference in the levels of expression and in the joint correlation, generating an interpretation contrary to the real one separately. Moreover, in the case of women, there is a clear need to consider the state of the menstrual cycle as well. Progesterone levels determined the relationship between miR-106b-5p levels and performance. This fact increases the relevance of hormone analysis for exercise planning in female athletes. Progesterone was described as a glucose metabolism modulator, including a reduction of GLUT4 in muscle, so this fact could determine our data in 500 m performance where the main energy substrates are carbohydrates (12). Moreover, higher levels of progesterone in our data seems to be a reducer in exercise 500 m performance as it was described by Lebrun et al. with a reduction of VO_2_max in luteal phase compared with follicular phase (13).

In acute response to exercise, c-miR-106b-5p was described as an acute responder to a VO_2_max test in amateur runners (4) and in a 2 h recovery period in a training session on elite cyclists (14); however, in training response, there were no changes on its level in elite cyclists (14) nor in amateur runners (5) in basal conditions, the same as it was observed in our data. Considering exercise performance, c-miR-106b-5p levels previous a maximal test was negatively associated with maximal aerobic speed in amateur runners (4), so considering our data this could add information to the possibility of miR-106b-5p as performance predictor. Moreover, in horses, the ones which had lower levels of mir-106b-5p were the ones which could end endurance races of 160 km, another data of miR-106b-5p as exercise performance predictor and the possibility of microRNAs as interspecies connector (15).

Talking about the *in silico* analysis, we had described that mir-106b-5p is determinant in energy metabolism and metabolite hydrolysis which is clearly in line with the previously described about glucose metabolism (16), and specifically, as a target of GLUT4 (17, 18). Inhibition of mir-106b-5p had an effect on increase glucose uptake and consumption (18). Taking together the fact that both progesterone and miR-106b-5p interacts with GLUT4, it could be propose as an explanation on mir-106b-5p exercise performance prediction (19). Moreover, *in silico* also reveals that miR-106b-5p is important on blood metabolism and also has a role on key pathways to angiogenesis such as hippo signaling pathway, mTOR, or FOXO (20–22). miR-106b-5p was previously validated as responsible of anti-angiogenesis role in endothelial cells, an overexpression determined a lower number of tubes (23). Thus, overall, within our group of athletes, the lower miR-106b-5p levels obtained in those with higher performance could be related to a higher angiogenesis and up-regulation of GLUT4 in both men and women.

Finally, our results could open the possibility of using miR-106b-5p as a predictive biomarker of physical performance and physical fitness. However, in female athletes the menstrual cycle must be taken into account to consider it as a biomarker.

## 5. Limitations

Some limitations should be remark. First, a larger number of athletes would have been desirable, but the whole number of the Spanish kayak Olympic team was considered in this study. Furthermore, the age range of men athletes was different from female athletes which could introduce a confounding factor. There is not much information about the effect of age on the c-miRNA profiles of humans, particularly in response to exercise. In this sense, although the study of Margolis et al. (24) described a differentiated c-miRNA response to acute exercise between young (22 ± 1 years) and old (74 ± 2 years) male volunteers, but our groups both are young and further investigation comparing men and women must be done. Finally, as our candidate c-miRNAs are highly expressed in a variety of cell types, their real source/s and target/s are not known, and it is out of the scope of this short study to go deeper than an *in silico* analysis. Mechanistic *in vitro* and *in vivo* studies are necessary to experimentally validate these findings.

## 6. Practical applications

This study highlights the need to further analyze the molecular response to exercise in men and women separately, and to consider the stage of the menstrual cycle in women as a relevant factor in this response and a determinant of performance. This will make it possible to move toward more personalized and, consequently, more effective recommendations for optimizing the performance and health of athletes.

## Data Availability

The raw data supporting the conclusions of this article will be made available by the authors, without undue reservation.
